# Protective Effect of Human-Neural-Crest-Derived Nasal Turbinate Stem Cells against Amyloid-β Neurotoxicity through Inhibition of Osteopontin in a Human Cerebral Organoid Model of Alzheimer’s Disease

**DOI:** 10.3390/cells11061029

**Published:** 2022-03-18

**Authors:** Jung Yeon Lim, Jung Eun Lee, Soon A Park, Sang In Park, Jung-Min Yon, Jeong-Ah Park, Sin-Soo Jeun, Seung Joon Kim, Hong Jun Lee, Sung Won Kim, Seung Ho Yang

**Affiliations:** 1 Department of Otolaryngology—Head and Neck Surgery, Seoul St. Mary’s Hospital, College of Medicine, The Catholic University of Korea, Seoul 06591, Korea; jylim8921@gmail.com (J.Y.L.); yjm0000@hanmail.net (J.-M.Y.); jahpark1@gmail.com (J.-A.P.); 2 Department of Neurosurgery, St. Vincent’s Hospital, College of Medicine, The Catholic University of Korea, Suwon 16247, Kyonggi-do, Korea; eunree@nate.com; 3 Department of Neurosurgery, Seoul St. Mary’s Hospital, College of Medicine, The Catholic University of Korea, Seoul 06591, Korea; bobby1127@naver.com (S.A.P.); ssjeun@catholic.ac.kr (S.-S.J.); 4 Institute of Catholic Integrative Medicine (ICIM), Incheon St. Mary’s Hospital, The Catholic University of Korea, Seoul 06591, Korea; parksi07@catholic.ac.kr; 5 Division of Pulmonology, Critical Care and Allergy, Department of Internal Medicine, Seoul St. Mary’s Hospital, College of Medicine, The Catholic University of Korea, Seoul 06591, Korea; cmcksj@catholic.ac.kr; 6 College of Medicine and Medical Research Institute, Chungbuk National University, Cheongju 28644, Korea; leehj71@chungbuk.ac.kr

**Keywords:** Alzheimer’s disease, amyloid-β peptide, human brain organoid, hNTSCs, osteopontin

## Abstract

The aim of this study was to validate the use of human brain organoids (hBOs) to investigate the therapeutic potential and mechanism of human-neural-crest-derived nasal turbinate stem cells (hNTSCs) in models of Alzheimer’s disease (AD). We generated hBOs from human induced pluripotent stem cells, investigated their characteristics according to neuronal markers and electrophysiological features, and then evaluated the protective effect of hNTSCs against amyloid-β peptide (Aβ_1–42_) neurotoxic activity in vitro in hBOs and in vivo in a mouse model of AD. Treatment of hBOs with Aβ_1–42_ induced neuronal cell death concomitant with decreased expression of neuronal markers, which was suppressed by hNTSCs cocultured under Aβ_1–42_ exposure. Cytokine array showed a significantly decreased level of osteopontin (OPN) in hBOs with hNTSC coculture compared with hBOs only in the presence of Aβ_1–42_. Silencing OPN via siRNA suppressed Aβ-induced neuronal cell death in cell culture. Notably, compared with PBS, hNTSC transplantation significantly enhanced performance on the Morris water maze, with reduced levels of OPN after transplantation in a mouse model of AD. These findings reveal that hBO models are useful to evaluate the therapeutic effect and mechanism of stem cells for application in treating AD.

## 1. Background

Alzheimer’s disease (AD) is an incurable, age-related neurodegenerative disorder that accounts for 50–70% of dementia cases worldwide [1]. AD, the most common form of dementia, is clinically identified as a progressive decline in neurocognitive function due to the deposition of neurotoxic proteins, such as extracellular senile amyloid β (Aβ) plaques and intracellular neurofibrillary tangles, causing neuronal and synaptic loss [2]. Although recent studies on the pathophysiological process or treatment of AD have been successfully conducted through studies using cell or animal models, even drugs with successful preclinical assessment have not been effective in reversing or slowing AD progression in clinical trials [3,4,5]. These disappointing outcomes are suggestive of limitations in translating therapies from animal models to humans [6]. Moreover, the complex neuropathology and mechanisms of AD have not been fully characterized.

Recently, three-dimensional (3D) cell models based on induced pluripotent stem cell (iPSC) technology have made it possible to study disease-specific phenotypes. In particular, iPSC-derived neurons from AD patients have been shown to exhibit the pathological features implicated in AD pathology in in vitro culture: extracellular or intracellular soluble Aβ accumulation or insoluble Aβ aggregation [7,8,9]. However, in studies involving differentiation of iPSC derived from AD patients, there is a limitation in that differentiated neuronal cells show features significantly different from those of neurons from clinical AD patients. In other cases, AD-free, iPSC–derived neurons could be induced to develop AD phenotypes by treatment with Aβ42 oligomers or Aβ42 inducers [10,11]. In this method, the pathophysiological features of AD can be detected, such as neuronal cytotoxicity and accumulation of Aβ in neuronal cells, but AD features such as extracellular Aβ plaque formation are absent.

Recent studies have demonstrated the potential of AD brain organoids to be used as a potential drug treatment platform. Raja et al. [12] demonstrated that familial AD patient-derived organoids exhibited AD-like pathophysiological features in a culture system, and treatment of the organoids with γ-secretase inhibitor compound E or a β-secretase inhibitor partially reversed amyloid and tau pathology [12]. Moreover, the suppression of tau phosphorylation was shown only after inhibition of Aβ species [12].

Brain organoid formation relies on the self-organizing ability of iPSCs to develop the organized structures of the brain while maintaining features of key brain developmental processes [13]. Chronic inflammation in the brain causes microglial activation, which disrupts neuron and synapse function and leads to the release of pro–inflammatory cytokines that directly contributes to neuronal damage and loss [14,15,16]. Several reports suggest that neuroinflammatory changes, including prolonged microglia activation, are key pathological components of AD [17,18,19]. It is reported that microglia can innately develop within a cerebral organoid model, and the response and transcriptome of these organoid-grown microglia to inflammatory stimulation closely mimics the response and transcriptome of adult microglia acutely isolated from postmortem human brain tissue [20]. Thus, 3D brain organoids could be a realistic 3D model to minimize the gap between 2D cell culture and animal models.

Stem-cell-based therapy is a promising, safe, and effective therapeutic strategy for several neurodegenerative diseases, including AD [21,22]. Although more elucidation is needed on the underlying mechanisms of stem-cell-based therapies, their rapid achievement has indicated therapeutic potential to reverse the neurodegeneration associated with AD and improve cellular and structural functions [23,24]. As several neurotrophic factors ameliorate the pathological features and neurocognitive deficits in animal models of Alzheimer’s disease, this therapeutic potential may be due in part to its neurosecretory effects [25]. Clinical trials using stem cells in patients with AD are currently in progress, but therapeutic effects have not yet been confirmed as expected based on preclinical studies. To ensure the therapeutic potential of stem cells in AD patients, several things need to be evaluated prior to a clinical trial, including the selection of a useful cell type and source for the treatment of AD and the identification of an important factor that can alleviate neuropathology.

Human-neural-crest-derived nasal turbinate stem cells (hNTSCs) are a valuable therapeutic source of adult stem cells for the treatment of neurological disorders, including AD. The hNTSCs can be obtained from inferior turbinate tissue that is removed during turbinate resection via minimally invasive collection procedures. Under proliferation conditions, hNTSCs showed a high proliferative capacity and strongly expressed the MSCs surface markers CD63, CD90, and CD105. Moreover, hNTSCs showed great adipogenic, osteogenic, chondrogenic, and neurogenic differentiation capability under the environmental conditions in vitro [26,27,28]. A recent study demonstrated the therapeutic potential of hNTSCs for use in neuronal regeneration in experimental acute ischemic stroke and AD and showed accelerated bone regeneration in a tibial defect model [28,29,30].

In the present study, we investigated the possibility of using human brain organoids (hBOs) as a potential stem cell therapy platform by evaluating the neuroprotective effect of hNTSCs against Aβ-induced toxicity in hBO culture and then analyzing the role of osteopontin (OPN) as a secretory protein involved in the therapeutic potential of hNTSCs against Aβ toxicity in hBOs and a 5 × FAD transgenic mouse model of AD.

## 2. Methods

### 2.1. Human Lung Tissue Dissociation and Generation of Human Induced Pluripotent Stem Cells (hiPSCs)

The study using human-normal-lung-tissue-cell-derived hiPSCs was conducted in accordance with the Institutional Review Board of the Catholic University of Korea Seoul St. Mary’s Hospital (KC18TNSI0033), informed consent regulations, and the Declaration of Helsinki. Before surgery, the participants provided written informed consent to participate in this study. Normal lung tissue was processed as soon as possible to minimize cell yield loss and maintain cell viability. Briefly, fresh tissue was washed in cold Hanks’ Balanced Salt Solution (HBSS, Thermo Fisher Scientific, Carlsbad, CA, USA) and minced into small (1 mm) pieces with a scalpel, followed by further dissociation using prewarmed digestion buffer containing 0.75 mL trypsin (10,000 U/mL; Sigma, Saint Louis, MO, USA) and 150 uL elastase (4.5 U/mL; Worthington, UK) and incubated for 45 min at 37 °C with shaking. The enzymatic activity was stopped using 40 mL inhibition solution (30 mL DMEM/F-12 (Invitrogen, Austin, TX, USA) and 10 mL fetal bovine serum (FBS, Thermo Fisher Scientific) and 1 mL DNase I (10,000 U/mL, Sigma)). Enzymatically digested samples were filtered through a 100, 70, and 40 μm cell strainer (BD Biosciences, San Jose, CA, USA) to remove cell debris and washed with Dulbecco’s Modified Eagle Medium (DMEM, Thermo Fisher Scientific). Cells were centrifuged at 350× *g* for 10 min, the supernatant was carefully aspirated, and the cell pellet was resuspended in 5 mL of red blood cell lysis buffer for 5 min at room temperature (RT). The reaction was quenched using 5 mL of DMEM, and the entire 10 mL of cell suspension was transferred to a 15 mL tube, followed by 10 min of centrifugation at 350× *g*. The supernatant was removed, the cell pellet was resuspended in a small airway epithelial cell growth medium bullet kit (SAGM, Lonza, Basel, Switzerland), and the cells were seeded in a culture plate and cultured for use in experiments.

The reprogramming process was conducted by introducing the reprogramming factors Oct4, Sox2, Klf4, and c-Myc using nonintegrative Sendai RNA viruses (CytoTune-iPS Sendai reprogramming Kit, Thermo Fisher Scientific) according to the manufacturer’s instructions. Briefly, one or two days before transduction, 5 × 10^4^ cells were plated in 12-well plates and cultured in DMEM supplemented with 1% (*v*/*v*) penicillin/streptomycin (antibiotics, Invitrogen) and 10% (*v*/*v*) FBS (Thermo Fisher Scientific) to reach 80% confluency on the day of transduction. Then, the medium was changed to new DMEM containing the CytoTune vectors at a 1:1:1:1 ratio. Plates were placed in a 37 °C, 5% CO_2_ incubator overnight. Following overnight incubation, the spent medium was replaced with fresh DMEM every other day for one week. Seven days after the introduction of the trait, the cells were harvested and grown on a vitronectin-coated plate with mTeSR (STEMCELL Technologies, Cambridge, MA, USA; Cat No Catalog #85857).

### 2.2. hiPSC Culture

The hiPSC cell line was used for all experiments. The hiPSCs were cultured under feeder-free culture conditions on dishes coated with Matrigel (Corning, Tewksbury, MA, USA; 354277) in mTeSR1 medium (Stem Cell Technologies, Cambridge, MA, USA; 85850). Passaging was performed enzymatically using Accutase (Thermo Fisher Scientific, A1110501) by splitting colonies in clumps every 6–7 days, followed by replating on vitronectin-coated dishes. The medium was changed every day.

### 2.3. Generation of hBOs

Organoids were generated using a STEMdiff Cerebral Organoid Kit (STEMCELL Technologies; 08570) assay following the manufacturer’s instructions. On day 0, hiPSCs at 90% confluence were dissociated into single cells using Accutase (5 min, 37 °C). The hiPSCs were resuspended in embryoid body formation medium with 10 μM Y27632 (Sigma-Aldrich Co., Seoul, Korea; Y503), a Rock inhibitor, and diluted to a concentration of 9 × 10^3^ cells per mL after centrifugation at 1000× *g* for 5 min. Then, 100 μL of cell suspension was distributed into each well of a low-attachment, 96-well, U-bottom plate (Corning) to form single EBs. The medium was changed every two days.

### 2.4. Transmission Electron Microscopy (TEM)

Samples were fixed in 4% (*w*/*v*) PFA for 1 h, washed with PBS, and then stained with 1.5% (*w*/*v*) osmium tetroxide overnight. The samples were then washed with PBS and stained with 1.5% (*w*/*v*) uranyl acetate solution; the insert membranes were then dehydrated with an acetone series and treated with propoxylene/epoxy resin (TAAB Laboratories Equipment Ltd., Berks, UK). After infiltration, it was again infiltrated overnight with 100% (*w*/*v*) epoxy resin. After trimming, the inserts were placed into silicone molds with fresh resin and cured at 65 °C for 48 h. Sectioning was performed using a UC6 ultramicrotome (Leica Microsystems, Wetzlar, Germany) or a Leica Ultracut UCT (Leica Microsystems). Ultrathin sections collected on grids (200 mesh) were examined by TEM (JEM 1010, T.E.M. Incorporated, Tokyo, Japan) operated at 60  kV, and TEM images were recorded by a CCD camera (SC1000, Gatan Inc., Pleasanton, CA, USA). The length on the electron micrograph was measured using GMS software (Gatan Inc., Pleasanton, CA, USA).

### 2.5. Generation of Aβ Oligomers

Aβ_1–42_ was purchased from Eurogentec (AnaSpec, Fremont, CA, USA; AS-64129-1), and oligomeric amyloid was prepared as described in Dahlgren et al. [31]. Briefly, Aβ lyophilized with a hexafluoroisopropanol film was dissolved in dry dimethyl sulfoxide (Sigma-Aldrich Co., Seoul, Korea) to obtain a concentration of 1 mM and sonicated for 180 s. Ham’s F-12 (Invitrogen, Carlsbad, CA, USA) was then added to a final concentration of peptide 0.1 mM, and the samples were rotated on a rotary shaker at 4 °C for 7 days.

### 2.6. Human-Neural-Crest-Derived Nasal Turbinate Stem Cell (hNTSC) Culture

The study procedure utilizing hNTSCs was performed in accordance with the Institutional Review Board of the Catholic University of Korea Seoul St. Mary’s Hospital (KC08TISS0341), informed consent regulations, and the Declaration of Helsinki. The participants provided written informed consent prior to surgery to participate in this study. The hNTSCs were isolated from discarded nasal inferior turbinate tissue from human patients who underwent partial turbinectomy, as previously described [26,27]. The isolated tissue was washed with saline and PBS (Thermo Fisher Scientific), cut into small pieces, plated on a culture dish, and a sterile glass cover slide was placed over the tissue. Tissues were cultured in α-minimum essential medium (α-MEM, Thermo Fisher Scientific) supplemented with 1% (*v*/*v*) penicillin/streptomycin (antibiotics, Invitrogen) and 10% (*v*/*v*) FBS (Thermo Fisher Scientific) at 37 °C in a humidified atmosphere containing 5% (*v*/*v*) CO_2_. The culture medium was changed every two days for 3 weeks of incubation. The glass cover was removed, and cells isolated from the tissue were harvested using 0.25% trypsin in 1 mM EDTA solution. The hNTSCs were expanded in four to five passages for use in experiments. The hNTSCs showed a rapid expansion during the culture and an ability to differentiate into multiple cell types in vitro [30].

### 2.7. Human Neural Stem Cell (hNSC) Culture

HB1F3 is a commercially available hNSC line kindly given to us as a gift by Dr. Hong Jun Lee (Chungbuk National University, Korea) [32].

The cells were cultured in Iscove’s Modified Dulbecco’s Medium (IMDM, WELGENE Inc., Gyeongsan, Korea; LM004-01) supplemented with 1% (*v*/*v*) penicillin/streptomycin (antibiotics, Invitrogen) and 10% (*v*/*v*) FBS (Thermo Fisher Scientific) at 37 °C in a humidified atmosphere containing 5% (*v*/*v*) CO_2_. HB1F3 cells were expanded for use in experiments.

#### Culture and Treatment

The hBOs were cultured in maturation medium. The hNTSCs (1 × 10^4^ cells) were cocultured in the upper chamber of a Transwell device (Merck Millipore, Burlington, MA, USA) with hBOs exposed to Aβ_1–42_ oligomers for 72 h in a 5% (*v*/*v*) CO_2_ incubator. For siRNA transfection, hNSCs (5 × 10^4^ cells) were plated in 24-well culture plates and maintained in growth medium. Twenty-four hours after plating, the cells were transfected with stealth RNAi oligonucleotides directed against the OPN sequence using Lipofectamine 3000 transfection reagent (Thermo Fisher Scientific; L3000001) and Opti-MEM (Thermo Fisher Scientific) medium. OPN oligonucleotides were obtained from Life Technologies Inc. (Santa Cruz Biotechnology Inc., Dallas, TX, USA; SC-23927) and were used at a final concentration of 80 pmol. After 4 h, fully supplemented growth medium was added to the plate, and the cells were maintained for 18 h. Then, the medium was replaced with 0.1% (*v*/*v*) FBS-containing medium, and the cells were incubated for an additional 24 h in the presence of 10 μM Aβ_1–42_ oligomers. Cell viability test was performed using a 3-(4,5-dimethylthiazol-2-yl)-2,5-diphenyltetrazolium bromide (MTT)-based cytotoxicity assay (Sigma-Aldrich Co., Seoul, Korea; M2128). Absorbance was measured at a wavelength of 590 nm using a microplate reader (Molecular Devices Corporation, Sunnyvale, CA, USA). Each experimental sample was examined in duplicate at least three times in quantitative RT-PCR.

### 2.8. Quantitative RT-PCR

Total RNA isolation from each sample was performed using Easyblue (iNtRON Biotechnology, Seongnam-si, South Korea) in accordance with the manufacturer’s protocol. One microgram RNA samples were used to generate cDNA with an iScript cDNA synthesis kit (Bio-Rad, Hercules, CA, USA). The qPCR was performed in triplicate on a real-time PCR system (Bio-Rad) using iQ SYBR Green Supermix (Bio-Rad) and 3.2 ng of RNA equivalents per 10 μL reaction. The cycling conditions were 40 cycles of 2 min at 95 °C, 15 s at 95 °C, and 60 s at 60 °C. Quantitative PCR was performed using a SYBR Green PCR mixture (Bio-Rad) according to the manufacturer’s instructions. RT-PCR for the 5′ coding region was performed with primers specific for Tubulin β-III (forward primer 5′-TCAGCGTCTACTACAACGAGGC-3′ and reverse primer 5′-GCCTGAAGAGATGTCCAAAGGC-3′), NeuN (sense primer 5′-TACGCAGCCTACAGATACGCTC-3′ and reverse primer 5′-TGGTTCCAATGCTGTAGGTCGC-3′), GFAP (forward primer 5′-CTGGAGAGGAAGATTGAGTCGC-3′ and reverse primer 5′-ACGTCAAGCTCCACATGGACCT-3′), Nestin (sense primer 5′-TCAAGATGTCCCTCAGCCTGGA-3′ and reverse primer 5′-AAGCTGAGGGAAGTCTTGGAGC-3′), and GAPDH (sense primer 5′-GTCTCCTCTGACTTCAACAGCG-3′ and reverse primer 5′-ACCACCCTGTTGCTGTAGCCAA-3′). The results were analyzed using Bio-Rad CFX Manager Version 2.1 software.

### 2.9. Cytokine Antibody Array

The cytokine secretion profile was examined by a cytokine antibody array human cytokine array C5 kit consisting of a total of 80 different cytokine antibodies spotted in membrane (Ray Biotech Inc., Norcross, GA, USA; AAH-CYT-5-8). The conditioned media from cultured human brain organoids with or without coculturing of hNTSCs in the presence of Aβ_1–42_ oligomers was collected for use in experiments. Array membrane was incubated with conditioned media and further incubated with biotin-conjugated antibody cocktail. The membrane was washed and incubated with HRP-conjugated streptavidin and developed using enhanced chemiluminescence detection reagents provided in the kit. Array images were scanned into a computer, and optical density was quantified in Image J by measuring the mean gray value.

### 2.10. ELISA Assay

Human osteopontin (OPN) in conditioned medium from cultured hBOs with or without coculture with hNTSCs in the presence of Aβ_1–42_ oligomers was quantified by ELISA kits according to the manufacturer’s instructions (R&D Systems, Inc., Minneapolis, MN, USA; DOST00). Human Aβ42 was quantified in AD mouse brain tissue homogenates by ELISA kit (Invitrogen, KHB3441) according to the manufacturer’s instructions. For analysis of soluble Aβ42, brain tissues were homogenized in ice-cold RIPA buffer (Thermo Fisher Scientific) containing protease inhibitors (GenDEPOT Inc., Barker, TX, USA) and sonicated. The homogenates were centrifuged at 20,000× *g* for 20 min at 4 °C, and the supernatant was analyzed as a soluble fraction.

### 2.11. Cell Transplantation

The in vivo study was conducted as previously described [30]. Mice expressing five mutants of human AβPP and PS1 (5 × FAD) (16 weeks of age, male; The Jackson Laboratory, Bar Harbor, ME, USA) was used according to institutional guidelines under conditions approved by the Animal Care and Use Committee of The Catholic University of Korea. The mice used in the experiment were divided into three injection groups (8 mice per group): (1) wild-type (WT) mice injected with PBS, (2) transgenic (Tg) mice injected with PBS, and (3) Tg mice injected with hNTSCs. Sixteen-week-old WT or Tg mice were anesthetized with ketamine (50 mg/kg; Zoletil, Virbac Laboratory, Carros, France) and xylazine (10 mg/kg; Rompun, Bayer, Seoul, Korea). Then 3 μL of PBS or cell suspension (1 × 10^5^) was injected bilaterally into the dentate gyrus of the hippocampus with a Hamilton syringe (26-gauge needle, Hamilton Company, Reno, NV, USA) using a microinfusion pump (KD Scientific, Holliston, MA, USA) in a stereotaxic apparatus. The injection rate of the cell suspension was 0.5 μL/min. The surgery was performed with aseptic technique, and antibiotics (gentamicin, 5 mg/kg SC, Shin Poong Co. Ltd., Seoul, Korea) and pain relief (ketoprofen, 5 mg/kg SC, SDC Pharm., Seoul, Korea) were administered to prevent infection before surgery.

### 2.12. Immunohistofluorescence Staining

The expression of Tubulin β-III (1:500, Biolegend, San Diego, CA, USA; 801201), Nestin (1:500, Santa Cruz Biotechnology Inc., SC-23927), GFAP (1:500, Merck Millipore; Burlington, MA, USA., AB5804), and NeuN (1:200, Merck Millipore, ABN78) in cultured human brain organoids was determined by immunofluorescence staining. The human brain organoid was fixed with 4% (*w*/*v*) PFA, and OCT-embedded human brain organoid tissue was cut (8 μm) using a freezing microtome (Leica Camera, Wetzlar, Germany). Tissue sections were blocked with 1% (*w*/*v*) normal goat serum (Jackson ImmunoResearch Laboratories, Inc., West Grove, PA, USA) and then incubated with primary anti-Tubulin β-III (1:500, Biolegend; 801201), anti-Nestin (1:500, Santa Cruz Biotechnology Inc.; SC-23927), or anti-GFAP (1:500, Merck Millipore; AB5804) antibodies and incubated with goat anti-mouse or rabbit Alexa Fluor 488 or 546 antibodies (1:1000; Molecular Probes, Eugene OR, USA, www.thermofisher.com). The nuclei were labeled with DAPI (1:1000, Sigma-Aldrich Co.), and fluorescence was observed using a Zeiss LSM510 confocal microscope (Carl Zeiss).

Apoptotic cells in hBO were visualized using a terminal deoxynucleotidyl transferase dUTP nick end labeling (TUNEL) assay kit (Merck Millipore; 11093070910) developed using Cy3-conjugated streptavidin (Jackson ImmunoResearch Laboratories, Inc., West Grove, PA, USA). In tissue sections, nuclei were stained with DAPI (1:1000, Sigma-Aldrich), and fluorescence was observed using a Zeiss LSM510 confocal microscope (Carl Zeiss).

For immunohistochemistry in mouse brain tissue, animals were sacrificed at 7 weeks after cell transplantation. The mice were anesthetized using ketamine (50 mg/kg; Zoletil) and xylazine (10 mg/kg; Rompun) and then perfused with 4% paraformaldehyde (Biosesang, Seongnam, Republic of Korea). To analyze Aβ plaques and target molecule expression in brains of 5 × FAD mice, brain tissues were cut using a frozen microtome (Leica Camera, Wetzlar, Germany). Prepared tissue sections were incubated with the following primary antibodies: anti-beta amyloid (6E10, 1:100; BioLegend, 803002), anti-Iba-1 (1:500; Wako, Osaka, Japan), anti-NeuN (1:200, Merck Millipore, ABN78), and anti-human nuclear antigen (HuNu, 1:100, Merck Millipore, MAB1281). The tissue nuclei were stained using DAPI (1:1000, Sigma-Aldrich), and then fluorescence was observed with a Zeiss LSM510 confocal microscope (Carl Zeiss).

### 2.13. Western Blots

To analyze protein expression in the brains of 5 × FAD mice, half of the brain tissues at 7 weeks after transplantation were homogenized in RIPA buffer (Thermo Fisher Scientific) containing protease inhibitors (GenDEPOT Inc., Barker, TX, USA) and sonicated. The homogenates were centrifuged at 20,000× *g* for 20 min at 4°C, and the supernatant was used for OPN protein analysis. For Western blots, protein samples were loaded onto 4–12% (*w*/*v*) or 12% (*w*/*v*) Bis-Tris Protein Gels (Thermo Fisher Scientific) and then transferred to a polyvinylidene difluoride (PVDF) membrane (Roche, Mannheim, Germany). The membrane was incubated with primary anti-OPN (1:500, Santa Cruz Biotechnology Inc., Dallas, Texas, USA; SC21742) and anti-β-actin (1:1000, Santa Cruz Biotechnology; SC47778) antibodies. The membrane was incubated with horseradish peroxidase-conjugated secondary antibodies and developed using enhanced chemiluminescence detection reagents (Thermo Fisher Scientific).

### 2.14. Behavioral Test

After 6 weeks of cell transplantation, spatial working memory and learning was evaluated by the Morris water maze (MWM) trial test. The MWM trial was conducted after release of a nontoxic white colorant into a circular pool (1.5 m in diameter) filled with opaque water (25 ± 1 °C) as previously described [30,33,34]. Before starting the trial, the mice were allowed to get used to the test environment through free swimming for 60 s in the pool, and then, after platform (10 cm in diameter) was exposed 1 cm above the water surface, the mice were allowed to swim for 60 s, and then placed on the platform for 20 s. The next day, the platform was placed 1 cm below the water surface level and the mice were trained to find the hidden platform through 3 trials per day for 8 days. Each trial was performed with starting the mouse in a different position in the quadrant where the platform was not placed, and the mice were allowed to search for the platform for up to a maximum of 60 s. After the trial, the mice were dried with a towel and placed in a warm cage to maintain body temperature. The trial was conducted at a similar time to minimize experimental variables. A probe trial was performed on day 9 to assess memory retention. During the probe trial, the mice were released into the pool directly opposite the location of the target platform and allowed to swim freely for 60 s. All mice used in the trial were monitored automatically and the time the mice stayed in each quadrant (zones 1–4) was recorded using the Smart 3.0 Video Tracking System (Panlab, S.L., Barcelona, Spain).

### 2.15. Quantification and Statistical Analysis

All data are presented as the mean (SD) of at least 3 independent experiments. ANOVA with a post hoc Tukey multiple-comparison test was used to determine whether differences between groups were statistically significant. Statistical differences between two different samples were determined with Student’s t-test. A probability value <0.05 was considered significant. For quantification of TUNEL-positive cells in brain organoid, cells were counted in 4 randomly selected nonoverlapping regions per section (four organoids per group). For quantification of HuNu- and NeuN-positive cells in brain tissue of 5 × FAD mice, cells were counted in three or four randomly selected, nonoverlapping and similar regions per section (four animals per group). Stained cells were counted by using Image-Pro Plus software (Media Cybernetics, Inc., Rockville, MD, USA, http://www.mediacy.com). To analyze Aβ plaque areas in brain tissue, immunofluorescence-positive regions in the brain sections were analyzed by using ZEN imaging software (Carl Zeiss).

## 3. Results

### 3.1. Characteristics of hiPSC-Derived hBOs

**Figure 1:**
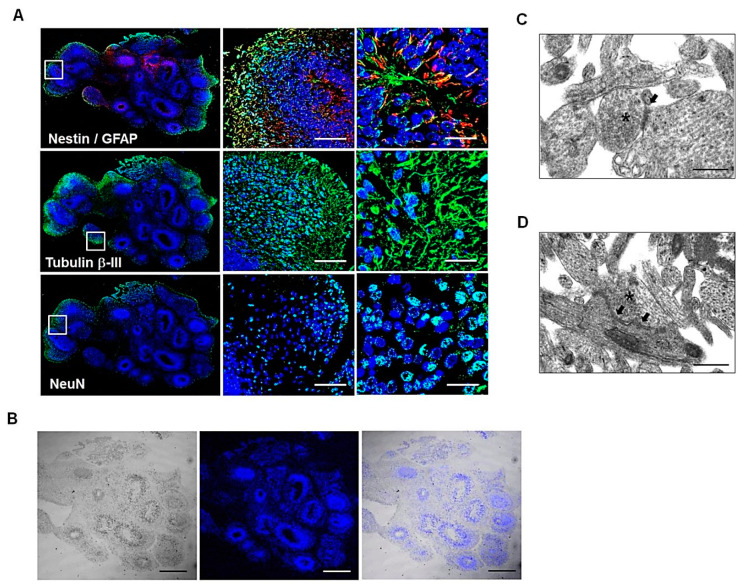
Characterization of hBOs in culture. (**A**) Confocal microscopy images of hBOs stained with antibodies against Nestin, GFAP, Tubulin β-III, and NeuN at day 60 in differentiation medium. Scale bars: 50 μm or 20 μm. (**B**) Confocal fluorescent image overlaid on the DIC image of hBO. Scale bar: 500 μm. (**C**,**D**) TEM images of cortical organoids showing synaptic clefts (head arrows) and synaptic vesicles (asterisks). Scale bar: 500 nm. All images are representative of two or three independent experiments.

After culturing hiPSCs under conditions that promote 3D neuroectoderm differentiation for 60 days, cerebral organoids form. Immunohistochemical analyses demonstrated the presence of different neuronal cell types in hBOs. Ventricle-like cavities of hBOs showed the expression of neuronal protein markers, such as β-III tubulin and NeuN, indicating the differentiation and maturation of newly formed neurons, and the neuroepithelial stem cell marker Nestin. Immunostaining for the intermediate filament protein GFAP showed the existence of glial cell types in hBOs (Figure 1A,B). Moreover, ultrastructure analysis by transmission electron microscopy (TEM) demonstrated the presence of typical synaptic structures showing synaptic vesicles and electron-dense synaptic contact sites (Figure 1C,D, asterisks and arrowheads, respectively).

### 3.2. Aβ_1–42_ Induces Neurotoxic Activity in hBO Culture

**Figure 2:**
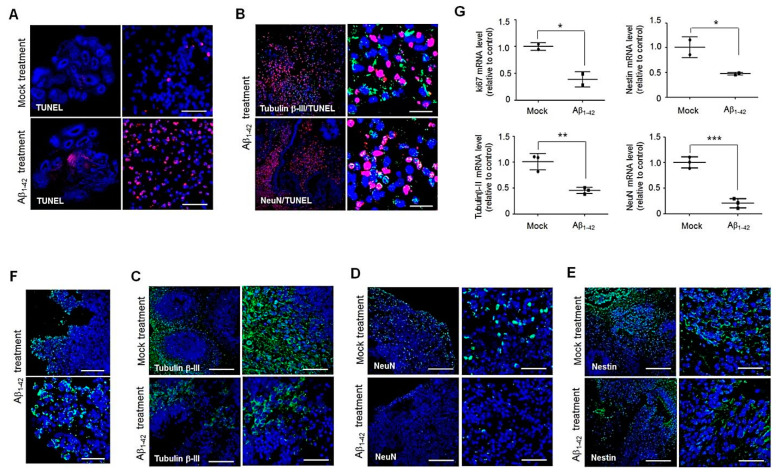
Histological analysis of hBOs cultured in the presence of Aβ_1–42_. (**A**–**E**) The hBOs were cultured in the absence or presence of 10 μM Aβ_1–42_ for 72–80 h. Confocal microscopy images of hBOs after staining OCT-embedded sections with a TUNEL assay kit (red) or with antibodies against tubulin β-III tubulin, NeuN, and Nestin (green). Nuclei were labeled with DAPI (blue). Scale bars: 50 μm, 20 μm, or 200 μm. (**F**) Confocal microscopy images of hBOs cultured in the presence of 10 μM Aβ_1–42_ after staining of OCT-embedded sections with an antibody against 6E10 to detect Aβ deposition (green). Nuclei were labeled with DAPI (blue). Scale bars: 200 μm or 50 μm. (**G**) Ki67, Tubulin β-III tubulin, Nestin, and NeuN mRNA levels were measured at 72–80 h after culture by quantitative real-time PCR in hBOs cultured with or without Aβ_1–42_. The GAPDH gene was used as a control. The values shown are the mean (SD). The significance of differences between two different samples was determined with Student’s *t*-test. * *p* < 0.05, ** *p* < 0.01, *** *p* < 0.001. All data are representative of two or three independent experiments.

Here, we examined whether neurotoxicity of Aβ, seen in the AD brain, is also observed in brain organoids. We used oligomeric Aβ_1–42_ to induce neuronal cell death in hBO cultures and showed that there was greater apoptotic cell death on day 4 of incubation with 10 μM oligomeric Aβ_1–42_ than in hBOs cultured in the absence of Aβ_1–42_ (Figure 2A). Moreover, some of the TUNEL-positive cells were double-positive for the neuronal markers Tubulin β-III and NeuN (Figure 2B), suggesting that the dying cells in the hBOs were neuronal cells. We then examined the expression of the neuronal markers Tubulin β-III, NeuN, and Nestin by immunofluorescence staining in hBOs cultured in the presence or absence of Aβ_1–42._ Confocal microscopy images showed that treatment with Aβ_1–42_ reduced the expression of neuronal markers compared with that in hBOs cultured in the absence of Aβ_1–42_ (Figure 2C–E). The expression levels of neuronal markers decreased significantly (by approximately 50%) in hBOs cultured in the presence of Aβ_1–42_ compared with hBOs cultured in the absence of Aβ_1–42_ (Figure 2G). At 4 days after culture, we detected Aβ_1–42_ expression in hBOs treated with Aβ_1–42_; previous neuronal cell death could be explained by treatment with Aβ_1–42_ (Figure 2F).

### 3.3. hNTSCs Inhibit Aβ_1–42_ Neurotoxic Activity in hBO Culture

**Figure 3:**
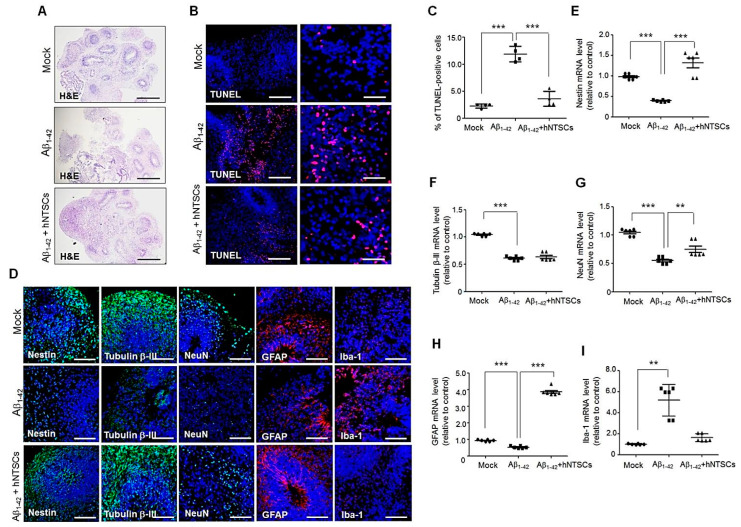
Histological analysis of hBOs cultured in the presence of Aβ_1–42_ with hNTSC coculture. (**A**) H&E images of hBOs cultured in the absence or presence of 10 μM Aβ_1–42_ with hNTSC coculture for 72–80 h after staining of OCT-embedded sections. Scale bar: 200 μm. (**B**) Confocal microscopy images of hBOs after staining OCT-embedded sections with a TUNEL assay kit (red). The hBOs were cultured in the absence or presence of 10 μM Aβ_1–42_ with hNTSC coculture for 72–80 h. Scale bars: 200 μm, 50 μm. (**C**) TUNEL-positive cells were counted in hBOs cultured in the absence or presence of 10 μM Aβ_1–42_ with hNTSC coculture for 72–80 h. The values shown are the mean (SD). For nonparametric multiple comparison tests, one-way ANOVA was used to determine whether differences between groups were statistically significant. *** *p* < 0.001. (**D**) Confocal microscopy images of hBOs cultured in the absence or presence of 10 μM Aβ_1–42_ with hNTSC coculture for 72–80 h after staining of OCT-embedded sections with antibodies against Nestin, Tubulin β-III, NeuN (green), GFAP (red), and Iba-1 (red). Nuclei were labeled with DAPI (blue). Scale bars: 200 μm or 20 μm. (**E**–**I**) Nestin, Tubulin β-III, NeuN, GFAP, and Iba-1 mRNA levels were measured by quantitative real-time PCR after 72–80 h of culture in hBOs cultured in the absence or presence of 10 μM Aβ_1–42_ with hNTSC coculture. The GAPDH gene was used as a control. The values shown are the mean (SD). For nonparametric multiple comparison tests, one-way ANOVA was used to determine whether differences between groups were statistically significant. ** *p* < 0.01,*** *p* < 0.001. All data are representative of two or three independent experiments.

To investigate whether hNTSCs protect neuronal cells against Aβ toxicity in hBO culture, we treated hBOs with oligomeric Aβ_1–42_ with or without hNTSC coculture for 72–80 h. The morphology of hBOs was observed with hematoxylin and eosin (H&E) staining. After 72–80 h of culture, staining showed that, compared with culture in the absence of Aβ_1–42__,_ treatment with Aβ_1–42_ induced cell death in hBOs. However, cell death was suppressed greatly by hNTSC coculture; many cells and structures were maintained in the hBOs (Figure 3A). Moreover, TUNEL staining showed that treatment with Aβ_1–42_ greatly increased the number of apoptotic cells in hBO culture compared with that in the control hBOs. After coculture with hNTSCs in the presence of Aβ_1–42_, a significant decrease in the number of TUNEL-positive apoptotic cells was observed in hBOs (Figure 3B,C). The average percentages of TUNEL-positive cells in the control hBOs and hBOs cultured in the presence of Aβ_1–42_ with or without hNTSC coculture were 2.3 (0.4%), 11.9 (1.4%), and 3.6 (1.4%), respectively (Figure 3C). Moreover, confocal microscopy images after immunofluorescence staining showed that treatment with Aβ_1–42_ reduced the expression of the neuronal markers Nestin and Tubulin β-III compared with that in the control hBOs. After coculture with hNTSCs in the presence of Aβ_1–42_, the expression of neuronal markers was observed to be as high as that in control hBOs. Moreover, confocal microscopy images showed that treatment with Aβ_1–42_ increased the expression of inflammatory microglial marker Iba-1 compared with control hBOs, which was suppressed by hNTSC coculture under Aβ_1–42_ exposure (Figure 3D). Quantitative RT-PCR showed that the expression levels of neuronal markers in hBOs cocultured with hNTSCs in the presence of Aβ_1–42_ were higher than hBOs cultured in the presence of Aβ_1–42_ without hNTSC coculture (Figure 3E–I).

### 3.4. Identification of OPN as a Functional Factor for hNTSC-Mediated Neuroprotection against Aβ_1–42_ Toxicity in hBO Culture

**Figure 4:**
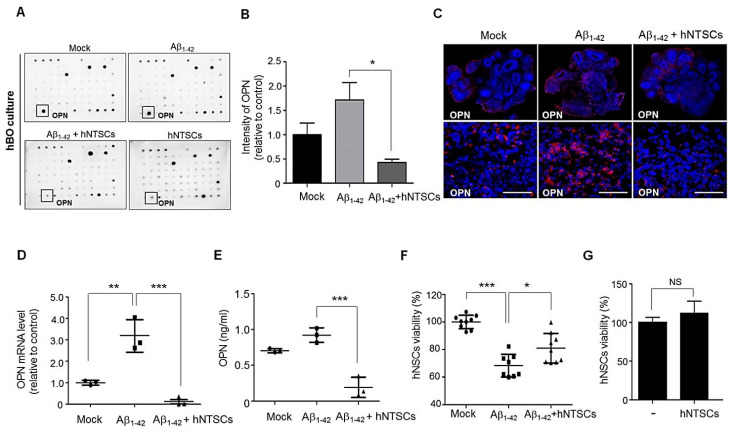
Cytokine analysis to identify a protein that is downregulated in the medium from hBOs cultured in the presence of Aβ_1–42_ with hNTSC coculture. (**A**) Medium was collected from hBOs and hNTSCs cultured alone, hBOs cultured in the presence of Aβ_1–42_, and hBOs cultured in the presence of 10 μM Aβ_1–42_ with hNTSC coculture for 72–80 h. The cytokine secretion profile was analyzed by incubating the array membrane with the medium from hBO culture. The box shows downregulated proteins in the medium from hBOs cultured in the presence of Aβ_1–42_ with hNTSC coculture. (**B**) The intensity of OPN spots in the array membrane was measured on the basis of grayscale levels. The values shown are the mean (SD). For nonparametric multiple comparison tests, one-way ANOVA was used to determine whether differences between groups were statistically significant. * *p* < 0.05. (**C**) Confocal microscopy images of hBOs cultured in the absence or presence of 10 μM Aβ_1–42_ with hNTSC coculture for 72–80 h after staining of OCT-embedded sections with antibodies against OPN (red). Nuclei were labeled with DAPI (blue). Scale bar: 50 μm. (**D**) OPN mRNA expression was measured at 72–80 h after culture by quantitative real-time PCR in hBOs cultured in the absence or presence of 10 μM Aβ_1–42_ with hNTSC coculture. The GAPDH gene was used as a control. The values shown are the mean (SD). For nonparametric multiple comparison tests, one-way ANOVA was used to determine whether differences between groups were statistically significant. ** *p* < 0.01, *** *p* < 0.001. (**E**) Medium was collected from hBOs and hNTSC cultured alone; hBOs cultured in the presence of Aβ_1–42_; and hBOs cultured in the presence of 10 μM Aβ_1–42_ with hNTSC coculture for 72–80 h. The OPN levels of hBO in culture were analyzed by OPN ELISA. The values shown are the mean (SD). For nonparametric multiple comparison tests, one-way ANOVA was used to determine whether differences between groups were statistically significant. *** *p* < 0.001. (**F**) Cell viability of hNSCs cultured in the presence of Aβ_1–42_ with hNTSC coculture. The hNSCs (in the lower chamber of a Transwell unit) were cultured in the presence of 10 μM Aβ_1–42_ with or without hNTSC coculture for 24 h. The viability of hNSCs treated with 10 μM Aβ_1–42_ with or without hNTSCs coculture was measured by an MTT assay. The values shown are the mean (SD). For nonparametric multiple comparison tests, one-way ANOVA was used to determine whether differences between groups were statistically significant. * *p* < 0.05, *** *p* < 0.001. (**G**) The viability of hNSCs cocultured with hNTSCs for 24 h was measured by an MTT assay. All data are representative of two or three independent experiments.

To identify the secreted functional factors that were involved in the neuroprotective effects of hNTSCs against Aβ_1–42_ toxicity in hBO culture, we performed a cytokine array by using a human cytokine array kit with conditioned culture medium from hBOs cultured in the presence or absence of 10 μM oligomeric Aβ_1–42_, Aβ_1–42_-treated hBOs with hNTSC coculture, and hBOs cocultured with hNTSCs alone (Figure 4A). All spots in the array membrane were quantified by averaging the two different membranes for each sample, and the intensity value revealed an approximately 1.7-fold higher level of OPN in hBOs treated with Aβ_1–42_ than in control hBOs (Figure 4B). OPN has been studied in several pathological neurodegenerative conditions, including multiple sclerosis [35,36], Parkinson’s disease [37,38], and AD [39,40,41], where OPN expression increases after neuronal damage. Interestingly, hNTSC coculture resulted in approximately 4.0-fold lower levels of OPN than culture of hBOs with only Aβ_1–42_, supporting the neuroprotective effect of hNTSCs in hBOs cultured with Aβ_1–42_ exposure (Figure 4B). Moreover, confocal microscopy images of hBOs after immunofluorescence staining showed that treatment with Aβ_1–42_ greatly increased the expression of OPN compared with that in control hBOs, but OPN expression was decreased in Aβ_1–42_-treated hBOs with hNTSC coculture (Figure 4C). Quantitative RT-PCR showed that there was an approximately 2.8-fold higher level of OPN in hBOs treated with Aβ_1–42_ than in control hBOs, but the levels of OPN were lower in Aβ_1–42_-treated hBOs with hNTSC coculture than in control hBOs (Figure 4D). Moreover, OPN levels were observed to be approximately 5-fold lower in conditioned culture medium from Aβ_1–42_-treated hBOs with hNTSC coculture than in hBOs cultured in the presence of oligomeric Aβ_1–42_ without hNTSC coculture (Figure 4E). Next, we sought to determine whether the neuroprotective effect of hNTSCs against Aβ toxicity seen in hBO culture is also observed in human neural stem cell (hNSC) culture. The hNSCs were cocultured with hNTSCs in the presence of 10 μM oligomeric Aβ_1–42_ for 24 h. The reduction in cell viability was measured by the MTT assay, and significant cell death (30–40%) was observed in hNSCs treated with Aβ_1–42_; these effects were significantly suppressed by hNTSC coculture (Figure 4F). However, the MTT results indicated that hNTSCs alone did not affect cell viability (Figure 4G).

### 3.5. Suppression of OPN Expression Inhibits Aβ_1–42_ Neurotoxic Activity in Human Neural Stem Cell (hNSC) Culture

**Figure 5:**
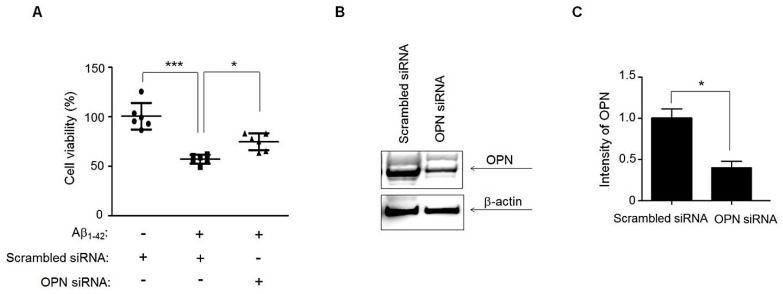
Cell viability of hNSCs cultured in the presence of Aβ_1–42_ with OPN siRNA treatment. (**A**) The viability of hNSCs transfected with OPN siRNA in the presence of 10 μM Aβ_1–42_ for 24 h was measured by an MTT assay. Mock-treated (lipofectamine only) cells or scrambled siRNA-treated cells were used as controls. The values shown are the mean (SD). For nonparametric multiple comparison tests, one-way ANOVA was used to determine whether differences between groups were statistically significant. * *p* < 0.05, *** *p* < 0.001. (**B**) Western blots of SDS-PAGE gels of hNSCs transfected with OPN siRNA in the presence of 10 μM Aβ_1–42_ using a primary anti-OPN antibody at 24 h after culture. β-actin was used as a loading control. (**C**) The values shown are the mean (SD). The significance of differences between two different samples was determined with Student’s *t*-test. * *p* < 0.05. All data are representative of two or three independent experiments.

To confirm that the neuroprotective effect of hNTSCs against Aβ toxicity is mediated by suppression of OPN expression, we examined whether the neurotoxicity of Aβ was suppressed in hNSC cultures in which OPN was silenced by siRNA. The hNSCs were transfected with OPN siRNA and incubated for an additional 24 h in the presence of 10 μM Aβ_1–42_ oligomers. The reduction in cell viability was measured by the MTT assay, and significant cell death (30%–35%) was observed in hNSCs treated with 10 μM oligomeric Aβ_1–42_; these effects were significantly suppressed by silencing of OPN via siRNA treatment compared to treatment of hNSCs with scrambled siRNA or the control treatment (Figure 5A). Western blots clearly showed that OPN-specific siRNA successfully downregulated OPN expression in hNSC cultures (Figure 5B,C).

### 3.6. Transplantation of hNTSCs Suppresses OPN Expression in the Brain of 5× FAD Mice

**Figure 6:**
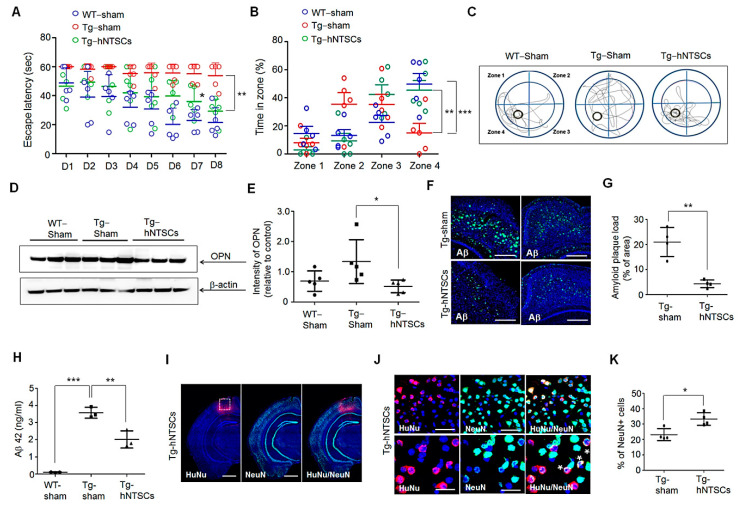
Effects of hNTSC transplantation on cognitive deficit and the expression of OPN in the brain of 5 × FAD mice. (**A**) The escape latency was the time in each group to find a hidden platform (up to a maximum of 60 s). For the multiple comparison tests, two-way ANOVA was used to determine whether group differences were statistically significant. * *p* < 0.05, ** *p* < 0.01. (**B**,**C**) Probe test images for remembering platform position in zone 4 in each group. Data were expressed as the percentage (%) of time spent in zones 1–4 within 60 s. (**D**) Western blots of proteins extracted from brain tissues of WT-sham, Tg-sham, and Tg-hNTSCs mice using a primary anti-OPN antibody. For the multiple comparison tests, two-way ANOVA test was used to determine whether group differences were statistically significant. ** *p* < 0.01, *** *p* < 0.001. (**D**) Western blots of SDS-PAGE gels of proteins extracted from brain tissues of WT-sham, Tg-sham, and Tg-hNTSCs mice using a primary anti-OPN antibody at 7 weeks after stem cell transplantation. β-actin was used as a loading control. (**E**) The values shown are the mean (SD). For nonparametric multiple comparison tests, one-way ANOVA was used to determine whether differences between groups were statistically significant. * *p* < 0.05. (**F**) Confocal microscopy images of Tg-sham and Tg-hNTSC brain sections with an antibody against 6E10 to detect Aβ deposition (green) at 7 weeks after stem cell transplantation. Nuclei were labeled with DAPI (blue). (**G**) Aβ plaque loads were quantified in the hippocampus and cortex of Tg-sham and Tg-hNTSCs (n = 4 per group). The values shown are the mean (SD). The significance of differences between two different samples was determined with Student’s *t*-test. ** *p* < 0.01. (**H**) Soluble Aβ42 levels in brain tissue lysates of WT-sham, Tg-sham, and Tg-hNTSCs at 7 weeks after stem cell transplantation (n = 3 per group). Values are the mean (SD). For the nonparametric multiple comparison tests, one-way ANOVA was used to determine whether group differences were statistically significant. ** *p* < 0.01, *** *p* < 0.001. (**I**) Confocal microscopy image of the brain of Tg-hNTSCs after double-staining of brain tissue sections with antibodies to HuNu (red) and NeuN (green). Nuclei were labeled with DAPI (blue). Scale bars: 200 μm. (**J**) A set of higher-amplification image of the part marked with the box in Figure 6I. HuNu–NeuN double-positive cells are marked with *. Scale bars: 100 μm, 500 μm. (**K**) NeuN-positive cells were counted in the cortex of Tg-sham and Tg-hNTSCs (n = 4 per group). Values are the mean (SD). The significance of differences between two different samples was determined with Student’s *t*-test. ** *p* < 0.01. All data are representative of two or three independent experiments.

To evaluate the effect of hNTSCs on enhancing learning and memory capacity, PBS or hNTSCs was injected into the brains of 16-week-old 5 × FAD transgenic mice (Tg-sham and Tg-hNTSCs). Morris water maze training was performed for normal WT mice (WT-sham), Tg-sham, and Tg-hNTSCs at 6 weeks after transplantation. During a training period of 8 day, both WT-sham and Tg-hNTSCs showed a progressive improvement in the ability to find the platform over time. However, Tg-sham mice showed significant cognitive impairment, and there were no significant differences at day 8 compared to day 1 of the training period (Figure 6A). Compared with Tg-sham mice, Tg-hNTSCs mice showed significant impairment on days 7 and 8 of the training period. By day 8, the average escape latencies of WT-sham, Tg-sham, and Tg-hNTSC mice were 21.5 (10.1 s), 53.9 (8.7 s), and 29.3 (8.4 s), respectively. Moreover, the probe trial showed that the Tg-hNTSC mice spent significantly more time in the target quadrant zone 4—similar to that of the WT-sham—when compared with the Tg-sham group (** *p* < 0.01). The average preference for the target quadrant of WT-sham, Tg-sham, and Tg-hNTSCs was 49.8 (17.0%), 14.9 (15.2%), and 45.4 (15.2%), respectively (Figure 6B,C). These results demonstrated that transplantation of hNTSCs significantly enhanced performance in the Morris water maze. Next, we sought to investigate whether transplantation of hNTSCs regulates the levels of OPN in AD brains. At 7 weeks after transplantation, brain extracts from WT-sham, Tg-sham, and Tg-hNTSC brains were subjected to Western blotting on SDS-PAGE gels. OPN levels, on average, were 1.9-fold higher in Tg-sham than in WT-sham. Notably, transplantation of hNTSCs significantly reduced the expression of OPN by approximately 2.5-fold compared with that in Tg-sham (Figure 6D,E), which confirms the reduced levels of OPN in hBOs with hNTSC coculture under Aβ_1–42_ exposure, as shown in Figure 4. Moreover, immunostaining of the brain with the Aβ-specific antibody 6E10 showed a greater reduction in Aβ plaque in the cortex and hippocampus of Tg-hNTSCs than Tg-sham (Figure 6F). The average Aβ plaque loads in the brains of Tg-sham and Tg-hNTSCs were 8.6 (3.6%) and 3.6 (3.1%), respectively (Figure 6G). ELISA of brain homogenates revealed that the levels of soluble Aβ42 were decreased significantly in the Tg-hNTSCs compared to the Tg-sham group (Figure 6H). Moreover, the transplanted human cells and neurons were analyzed by immunofluorescence staining for HuNu and NeuN. Seven weeks after transplantation, many human cells were detected around the stereotaxic injection site in the brains of Tg-hNTSC mice (Figure 6I,J). The average number of HuNu-positive cells around the injection site in the brains of Tg-hNTSCs was 315 (66.6 cells). The average percentages of NeuN-positive cells in the cortical regions of Tg-sham and Tg-hNTSCs were 23.2 (3.8%) and 31.6 (6.3%), respectively (Figure 6K). Some of cells in Tg-hNTSCs were double-positive for HuNu and NeuN (Figure 6H), suggesting that some human cells engrafted into the brain differentiated into neuronal cells in Tg-hNTSCs. These data support the conclusion that the protective effects of hNTSCs are mediated by OPN suppression, providing mechanistic proof for the effectiveness of hNTSC treatment.

## 4. Discussion

Regenerative therapy using stem cells has been regarded as a promising and safe approach for the restoration of altered or lost cellular functions. Although the underlying mechanisms of stem-cell-based therapy need more clarification, several preclinical studies have generated encouraging results [23]. There have been an increasing number of stem cell trials in patients with AD [42,43], but the great therapeutic effects expected based on preclinical studies have not been achieved.

Two-dimensional (2D) monolayer cultures are the most common and most traditional models used for in vitro research. Though 2D culture models have contributed to significant findings and advances in biological research on AD, these models have intrinsic limits on their validity as models of in vivo biology [7,9,44]. The limitations of 2D cultures triggered the development of in vitro 3D cell models capable of resembling the architecture, cellular function, and tissue conditions that more closely mimic the microenvironment observed in the human brain. These are important features in drug test platforms for the treatment of AD since environmental cues have profound effects on the properties, behaviors, and functions of cells, which may affect cellular responses to drugs [45,46]. There is no doubt that the use of animals in therapeutic medicine has contributed greatly to the development of human therapies. However, there are several limitations associated with the implementation of animal studies to elucidate the molecular mechanisms underlying complex disease features [47,48,49,50]. It is impossible to study tau pathology or early AD features in transgenic mice under AD pathophysiological conditions, which limits their potential use as suitable AD model systems.

Thus, 3D culture models of human cells or human tissues offer an alternative to natural tissue models for systemic experimentation, reducing the need for animals and permitting a more straightforward understanding of cause and effect in drug safety and efficacy assessments. The improvement of iPSC differentiation protocols led to the establishment of a 3D brain organoid with a self-organized structure resembling the complex human brain structure and distinguishable cell fate in the culture system [51,52,53]. Thus, many studies have focused on brain organoids as a valuable evaluation platform for the discovery of effective AD treatments.

In this study, we validated the feasibility of hBOs as a potential AD platform for hNTSC therapy for the treatment of AD. Prior to investigating the therapeutic potential of hNTSCs in AD hBO models, the characteristics of human iPSC-derived hBOs were analyzed by immunofluorescence staining and electron microscopy. Protocols for differentiating hiPSCs to form brain organoids were performed via EB generation in differentiation medium for approximately 60 days of culture. Immunostaining of hBOs showed the presence of different neuronal cell types, such as proliferating NSCs, differentiating and mature newly formed neurons, and glial cells in hBO culture. Moreover, electron microscopy of hBOs showed axon-like microtubules and astrocytes (Figure 1).

There are several protocols to express the AD phenotype in in vitro culture systems. Differentiation of EBs generated from hiPSCs from AD patients to form brain organoids has yielded the pathological features implicated in AD pathology in in vitro culture, including extracellular or intracellular soluble Aβ accumulation or insoluble Aβ aggregation [54]. Another way to establish a brain organoid model with an AD phenotype is based on chemical induction with Aβ_1–42_ oligomers or Aβ inducers in the culture of AD-free iPSC-derived brain organoids [10,11]. To generate an AD phenotype in normal brain organoids, we added 10 μM Aβ_1–42_ oligomers into the culture medium of hBOs for 72–80 h, and immunofluorescence imaging revealed greatly increased neuronal cell death and decreased neuronal cell numbers in hBOs treated with Aβ_1–42_ oligomers compared with control hBOs (Figure 2). Moreover, we detected Aβ_1–42_ expression only in hBOs treated with Aβ_1–42_ oligomers but not in control organoids; previous neuronal cell death could be explained by treatment with Aβ_1–42_ oligomers (Figure 2F). These data indicate that Aβ treatment could induce pathophysiological features of AD in hBOs, such as neuronal cytotoxicity and Aβ detection, as shown in Figure 2.

Stem cell therapy in AD models has demonstrated neuroprotective potential by modulating neuroinflammation, boosting survival signaling, enhancing endogenous hippocampal neurogenesis, and suppressing neuronal apoptosis [55,56]. The ability of transplanted stem cells to replace damaged neurons through neuronal differentiation and to stimulate the endogenous neuronal repair system through the secretion of therapeutically important neurotrophic factors or neuroprotective cytokines has been demonstrated [57,58,59]. Conveniently, hNTSCs can be easily isolated from inferior turbinate tissue through minimally invasive procedures [26,27]. Further, hNTSCs have shown a strong proliferation capacity and an ability to differentiate into multiple cell types in vitro [27,60]. Moreover, a recent study demonstrated the therapeutic potential of hNTSCs for use in neuronal regeneration in experimental acute ischemic stroke and in accelerated bone regeneration in a tibial defect model [28,29]. Here, treatment with Aβ_1–42_ induced neuronal cell death in hBO culture, but cell death was suppressed greatly by hNTSC coculture; H&E staining showed that many cells were retained and the structure was maintained in the hBOs cocultured with hNTSCs. Moreover, hNTSC coculture was shown to support neuronal cell survival after treatment with Aβ_1–42_ oligomers (Figure 3), suggesting that hNTSCs may be advantageous for treating AD in terms of their strong neuroprotective effect against Aβ toxicity.

Additionally, we examined how hNTSCs protect neuronal cells against Aβ neurotoxicity in hBOs. Interestingly, we identified a secreted protein, OPN, whose expression was increased in hBOs cultured in the presence of Aβ_1–42_ but decreased significantly by coculture with hNTSCs, as determined by cytokine analysis of conditioned medium (Figure 4). Aβ plaques and an inflammatory response in the brain are the main features of AD pathogenesis [61,62,63]. Chronic inflammation in the brain causes microglial activation, which disrupts neuron and synapse function and leads to secretion of inflammatory cytokines that can injure neurons directly [14,15,16]. OPN is a multifunctional, proinflammatory cytokine that is involved in degenerative processes in the nervous system, including AD [39,40,41], multiple sclerosis [35,36], and Parkinson’s disease [37,38]. OPN has been studied in several physiological and pathological conditions of the central nervous system, and its production is upregulated in response to either inflammation or injury [64]. In AD, several studies have shown an important role of inflammatory processes, such as microglial activation and local upregulation of acute-phase proteins, complement, cytokines, and other inflammatory mediators, mainly around amyloid plaques, especially in the early stages of the disease [55,65]. For this reason, it is important to identify the role of OPN in hNTSC therapy to ameliorate neuropathology during the treatment of AD. Coculture with hNTSCs greatly reduced OPN expression in human organoids cultured in the presence of Aβ_1–42_ concomitant with an increased number of neuronal cells (Figure 4). Moreover, suppression of OPN expression by silencing OPN via siRNA treatment inhibited Aβ_1–42_ neurotoxic activity in hNSC cultures (Figure 5), suggesting that the protective effects observed after hNTSCs treatment are mediated by OPN suppression. This result is important in that it is the first evidence that OPN plays an important role in stem cell treatment of Aβ neurotoxicity using a brain organoid platform. To further evaluate the role of OPN in AD pathology in vivo, we directly injected hNTSCs into the brains of 5 × FAD mice that overexpress five familial AD transgenes. Notably, our data demonstrated the relevance of OPN function for hNTSC therapy in vitro in brain organoids and in the mouse brain in the AD model. Tg-hNTSC mice showed significantly lower levels of OPN in the brain than Tg-sham mice at seven weeks after transplantation (Figure 6), with greatly enhanced performance on the Morris water maze; this could be explained by the transplantation of hNTSCs. Therefore, the use of hNTSCs to protect neuronal cells by suppressing OPN expression would be a valuable therapeutic strategy for the treatment of AD. This work is significant in that it is the first study to show the importance of OPN function in the treatment of AD by using stem cells in an in vitro brain organoid platform and in vivo animal experiments. Further functional analysis of OPN as a therapeutic target in the treatment of AD using stem cells is needed.

In the present study, we showed the feasibility of using hBOs as a potential stem cell therapeutic platform in AD treatment and demonstrated an important role of OPN in hNTSC therapy in AD brain organoid models and AD mouse models. The hNTSCs showed a great ability to protect neuronal cells against Aβ-induced neurotoxicity in an hBOs model in vitro. Notably, hNTSC treatment strongly suppressed the expression of OPN, which is involved in the inflammatory response in AD models in vitro and in vivo. Although many studies have recently attempted to use brain organoids for AD drug screening, this study is significant in that it is the first to use hBOs as a platform for stem cell therapy. Moreover, this study is significant in that it is the first to reveal the importance of OPN function in stem cell therapy using a brain organoid platform. The reliable evidence provided by these findings reveals that hBO systems are useful for evaluating the efficacy of stem cell therapy for the clinical translation of preclinical studies on AD.

## 5. Conclusions

This study showed the feasibility of using hBOs as a potential stem cell therapeutic platform for the treatment of AD by evaluating the efficacy of hNTSCs in an hBO model of AD and then analyzing the important role of OPN in hNTSC therapy in an AD hBO model and an AD mouse model. The hBOs were generated from hiPSCs, and different neuronal cell types, synaptic vesicles and electron-dense synaptic contact sites were present. Treatment with Aβ_1–42_ in hBO cultures induced neuronal cell death concomitant with decreased expression of neuronal markers. Coculture with hNTSCs greatly protected neuronal cells against Aβ-induced neurotoxicity in hBOs in vitro. Notably, hNTSC treatment clearly showed a protective effect on neuronal cells by suppressing the expression of OPN, which is involved in the inflammatory response, in in vitro and in vivo models of AD. Our findings reveal that hBOs are a useful platform to evaluate the efficacy and therapeutic mechanism of stem cell therapy for the treatment of AD.

## Data Availability

The datasets generated during and/or analyzed during the current study are available from the corresponding author on reasonable request.

## Abbreviations

Aβ_1–42_	Amyloid-β peptide
AD	Alzheimer’s disease
BO	Brain organoid
DAPI	4′,6-Diamidino-2-phenylindole
DMEM	Dulbecco’s modified eagle medium
FBS	Fetal bovine serum
HBSS	Hanks’ balanced salt solution
iPSC	Induced pluripotent stem cell
α-MEM	α-Minimum essential medium
NCSC	Neural crest-derived stem cell
NSC	Neural stem cell
NTSCs	Neural crest-derived nasal turbinate stem cells
PBS	Phosphate-buffered saline
OPN	Osteopontin
PFA	Paraformaldehyde
TUNEL	Terminal deoxynucleotidyl transferase dUTP nick end labeling
TEM	Transmission electron microscopy
3D	Three dimensional
TG	Transgenic
2D	Two-dimensional
WT	Wild-type
